# Platelet-Derived Granules and Extracellular Vesicles in Neurodegenerative Diseases: Neurovascular Mechanisms and Clinical Implications

**DOI:** 10.3390/cells15080692

**Published:** 2026-04-14

**Authors:** Bo Kyung Lee, Jae-Ryeong Yoo, Yi-Sook Jung

**Affiliations:** 1 Department of Pharmacy, Ajou University, Suwon 16499, Republic of Korea; pfiffer@ajou.ac.kr (B.K.L.); jryoo@ajou.ac.kr (J.-R.Y.); 2 Research Institute of Pharmaceutical Sciences and Technology, Ajou University, Suwon 16499, Republic of Korea

**Keywords:** platelet, extracellular vesicles, platelet granules, Alzheimer’s disease, biomarkers, neurodegenerative disease

## Abstract

**Highlights:**

**What are the main findings?**

- Platelet granule-derived mediators and extracellular vesicles cooperatively derive neurovascular dysfunction in neurodegenerative diseases.
- Distinct platelet secretory pathways—including α-granules, dense granules, and platelet-derived EVs—activate inflammatory, oxidative, and protein aggregation mechanisms in Alzheimer’s disease.

**What is the implication of the main finding?**

- Platelet-derived mediators represent promising biomarkers and therapeutic targets for early detection and intervention in neurodegenerative diseases.
- Integrating platelet secretion and EV signaling into a unified neurovascular framework provides a conceptual basis for future mechanistic and translational studies.

**Abstract:**

Platelets are increasingly recognized as multifunctional regulators that extend beyond hemostasis to actively engage in immunological regulation and neurovascular homeostasis. Platelets employ specialized secretory mechanisms, including granule-dependent release and extracellular vesicle (EV) shedding, to convey diverse bioactive mediators to vascular, immune, and neural cells. Growing evidence indicates that platelet-derived granules and EVs significantly influence the neurovascular unit, regulate inflammatory signaling, and modify neuronal function in both health and disease. In neurodegenerative disorders, particularly Alzheimer’s disease (AD), accumulating evidence suggests that platelet activation may be increased in neurodegenerative conditions, including AD, although the extent and causality of this activation remain under investigation. This review delineates the secretory apparatus of platelets and their mechanistic functions in intercellular communication, underscores platelet contributions to AD and other neurological disorders, and explores novel clinical prospects for biomarker development and therapeutic targeting based on platelet-derived EVs.

## 1. Introduction

### 1.1. Platelets in Hemostasis

Platelets are the primary effector cells in the hemostatic response to vascular injury, coordinating a complex series of molecular events that result in clot formation. Following endothelial disruption, platelets are the initial cells to attach to the exposed subendothelial matrix, swiftly converting the injured site into a microenvironment conducive to thrombin production. Thrombin, a serine protease, is generated through the convergence of the extrinsic and intrinsic coagulation pathways, which function synergistically to expedite clot formation [1]. The extrinsic pathway is triggered by the interaction of tissue factor and factor VII, which occurs following endothelial injury; conversely, the intrinsic pathway is activated by factor XII in the presence of phospholipids. Both pathways converge, ultimately leading to the assembly of membrane-associated enzyme complexes on the platelet surface, which significantly amplifies thrombin generation. Following its production, thrombin then retroactively activates platelets, initiating their activation and promoting the conversion of fibrinogen to fibrin, thus constructing the thrombus’s structural foundation. In this process, platelets serve not only as passive substrates but also as active regulators integrating diverse extracellular signals through numerous surface receptors. Key agonists, including thrombin, ADP, and thromboxane A_2_, activate platelets through G protein-coupled receptors, with thrombin being the most potent by interacting with PAR1 and PAR4, which couple to Gq, Gi, and G12/13 proteins [2]. Simultaneously, collagen exposed at the site of vascular injury interacts with the GPVI/Fc receptor complex, triggering tyrosine kinase-dependent signaling via ITAM-mediated recruitment of SYK [3,4]. The convergence of these signaling pathways ultimately results in the activation of integrin GP IIb/IIIa (αIIbβ3), which facilitates robust platelet–platelet adhesion via fibrin bridging [5,6]. In this meticulously orchestrated process, platelets serve as the pivotal agents of hemostasis—facilitating thrombin production, enhancing activation signals, and forming the stable thrombus that occludes the vascular injury.

### 1.2. Platelets as Immune Sentinels in Inflammation

In addition to their classical hemostatic roles, platelets serve crucial functions as immune sentinels during critical events such as vascular injury, infection, ischemia, and trauma. In these critical circumstances, they function as swift responders that detect biochemical danger signals and attach to damaged endothelium, commencing both hemostatic and inflammatory processes. Through the facilitation of rapid activation and aggregation, platelets augment thrombin synthesis and fibrin network formation, thus reinstating vascular integrity. Concurrently, they undertake immunomodulatory functions vital for survival in stressful conditions. Platelets exhibit Toll-like receptors (TLR2, TLR4, and TLR9), enabling them to directly identify microbial constituents and circulating pathogens [7,8]. Upon activation, they release antimicrobial peptides and microbicidal molecules, and in certain instances, internalize pathogens via phagocytosis, thereby directly contributing to host defense [9]. These actions establish platelets as initial effector cells that contain and eradicate pathogens prior to the complete recruitment of conventional immune cells [10].

Concurrently, platelets orchestrate a more extensive inflammatory response by enlisting and activating leukocytes. They enhance leukocyte activation and intercellular communication through the secretion of platelet factor 4 (PF4), RANTES, IL-1β, and CD40L. In neutrophils, platelet signals trigger the production of reactive oxygen species (ROS) and the secretion of proteolytic enzymes; in monocytes, they activate prothrombotic and inflammatory gene expression; and in lymphocytes, they facilitate B-cell class-switch recombination and augment cytotoxic T-cell activity [11,12]. This comprehensive platelet–leukocyte interaction establishes a rapid-response network that curtails infection dissemination and stabilizes vascular function during emergencies. Nevertheless, excessive or prolonged activation of this system can be detrimental, resulting in endothelial and tissue damage that may lead to sepsis, ischemic hepatitis, acute lung injury, and other inflammatory disorders [13]. Platelets serve as both protectors and potential enhancers of inflammation, sustaining a fragile balance between protective hemostasis and detrimental thrombosis. This dual function characterizes the nascent concept of immunohemostasis, in which platelets connect coagulation and immune defense as adaptable regulators of vascular homeostasis [14]. In acute emergencies, they concurrently initiate clot formation and regulate immune activation to maintain tissue integrity, while meticulously adjusting these responses to avert collateral damage [15]. The recognition of platelet-specific receptors, including CLEC-2 and TREM-like transcript-1, as regulators of these processes offers promising therapeutic targets for managing platelet activity during severe inflammatory or thrombotic responses [16,17]. Platelets should be regarded not merely as agents of hemostasis but also as protectors of vascular integrity and facilitators of immune coordination, essential for survival during physiological stress [15,18].

### 1.3. Platelet-Derived Extracellular Vesicles in Inflammation

In acute stress, platelets function as sensors and effectors, converting neuroendocrine and hemodynamic signals into cellular communication signals. Exposure to stress—be it psychosocial or physical—stimulates the sympathetic nervous system and the hypothalamic–pituitary–adrenal axis, resulting in increased levels of catecholamines and glucocorticoids. Hormonal and mechanical stimuli swiftly activate circulating platelets, inducing the release of EVs, specifically small extracellular vesicles (sEVs) and microvesicles originating from the platelet plasma membrane [19,20]. Platelet-derived extracellular vesicles (pEVs) constitute one of the most prevalent vesicle populations in peripheral blood and serve as a vital connection among the vascular, immune, and nervous systems during periods of stress [21]. pEVs encompass a varied array of bioactive molecules, including phosphatidylserine, integrins (notably α_IIb_β_3_), adhesion receptors (CD62P, CD41), enzymes, and small RNAs such as miRNAs, facilitating swift intercellular communication [19,22]. Upon release, they can engage with endothelial cells, leukocytes, and neural or glial cells, affecting vascular tone, permeability, and inflammatory signaling [23,24,25]. During acute physical stress, increased shear stress and catecholamine release facilitate platelet activation and a rise in pEVs that influence coagulation potential and endothelial function [26]. Conversely, psychosocial stress, marked by cortisol-mediated immune modulation, seems to trigger a more nuanced yet coordinated release of pEVs enriched with signaling molecules that regulate immune responsiveness and metabolic equilibrium [21].

pEVs are not simply by-products of platelet activation; they are dynamic messengers that transmit stress-specific information to remote tissues [19]. Surface integrins and tetraspanins enable selective uptake by target cells, while the RNA and protein cargo indicate both platelet activation status and systemic stress conditions [27,28]. Through these mechanisms, pEVs may engage in bidirectional communication between the brain and peripheral organs, augmenting the function of neuroendocrine mediators [29]. During acute psychosocial stress, pEVs may facilitate neurovascular coupling and behavioral regulation by transmitting stress-related miRNAs across the blood–brain barrier, while during physical exertion, they may coordinate metabolic adaptation between muscular and vascular systems [21,22,30,31,32].

Consequently, in the realm of acute stress, platelets expand their role significantly beyond hemostasis. By introducing EVs that reflect and adjust systemic physiological conditions, they contribute to the precise coordination of immuno-metabolic and vascular adaptation [19,32]. Examining the distinctions in platelet-derived vesicle signatures between psychosocial and physical stressors could offer significant insights into individual stress resilience and potentially identify novel biomarkers or therapeutic targets for stress-related disorders [33].

This review analyzes the novel functions of platelet-derived granule contents and EVs as critical mediators of neurovascular dysfunction in neurodegenerative disorders. We examine the impact of platelet secretory products and EV cargo on vascular integrity, neuroinflammation, and neuron-glia interactions, with a specific focus on Alzheimer’s disease (AD). Ultimately, we examine the clinical ramifications of platelet-derived factors as biomarkers and prospective therapeutic targets in neurodegenerative diseases. Although multiple studies have reported enhanced platelet activation markers in patients with neurodegenerative disorders, the degree to which platelet hyperactivation directly contributes to disease progression remains incompletely understood. Notably, functional evidence linking platelet activation to causal neurodegenerative processes is still limited, and many observations remain associative rather than mechanistic. These limitations highlight the need for standardized functional assays and longitudinal studies to better define the role of platelet activation in neurodegeneration.

The conceptual novelty of this review lies in establishing a unified neurovascular framework that integrates platelet granule secretion and EV-mediated signaling into a coordinated pathological axis. Unlike previous reviews that have examined platelet activation of EV biology as separate processes, this review proposes that platelet secretory pathways function as interconnected systems that operate across spatial and temporal scales. Specifically, this framework introduces three key conceptual advances. First, it defines platelet granule release and EV signaling as complementary mechanisms that generate both rapid local responses and sustained long-range communication within the neurovascular unit. Second, it conceptualizes platelet-derived mediators as systemic amplifiers that translate peripheral vascular activation into central neurodegenerative signaling through receptor-mediated and transcriptional pathways. Third, it highlights the convergence of granule-derived factors and EV cargo on shared pathological endpoints, including BBB disruption, neuroinflammation, and protein aggregation, thereby providing a mechanistic bridge linking vascular dysfunction to progressive neurodegeneration. By integrating these processes into a single mechanistic continuum, this review provides a conceptual platform for understanding how platelet-derived signaling contributes to neurovascular dysfunction and offers a framework for identifying novel therapeutic targets and biomarker strategies in neurodegenerative diseases.

## 2. Platelet Secretory Machinery and Intercellular Communication

**Figure 1:**
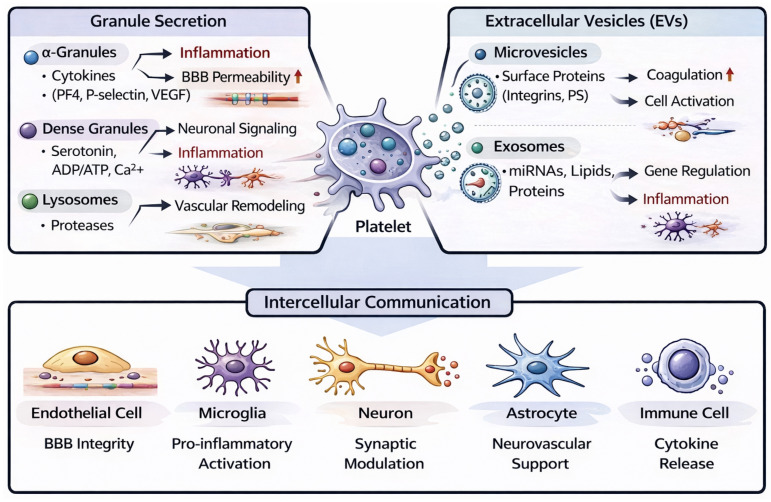
Platelet secretory systems and intercellular communication pathways.

Platelets have a specialized secretory apparatus that allows for a swift response to vascular injury and systemic stress [34,35,36]. Although devoid of a nucleus, platelets possess specific intracellular storage compartments and vesicle-producing pathways that enable the secretion of various bioactive mediators [37,38]. Platelet secretory mechanisms can be broadly categorized into two functionally and mechanistically distinct systems: granule-dependent secretion and extracellular vesicle (EV) release (Figure 1). Granule secretion originates from specialized intracellular storage organelles, including α-granules, dense granules, and lysosomes, which rapidly release pre-stored mediators through membrane fusion upon platelet activation. This process enables immediate and localized responses, such as platelet aggregation, vascular modulation, and leukocyte recruitment at sites of vascular injury. In contrast, EV release involves the generation of membrane-derived vesicles, including microvesicles formed by outward budding of the plasma membrane and exosomes originating from multivesicular bodies. Unlike granule secretion, which primarily supports acute local signaling, EVs function as mobile carriers that transport protein, lipids, and nucleic acids to distant target cells, thereby enabling sustained and long-range intercellular communication. These vesicles can circulate systemically and influence endothelial, immune, and neural cells beyond the initial site of platelet activation [39,40]. Recognizing these mechanistic and functional differences between granule-dependent secretion and EV-mediated signaling is essential for understanding how platelet-derived factors contribute differentially to vascular dysfunction, neuroinflammation, and progressive neurodegeneration (Table 1) [41,42].

### 2.1. Granules as Expedited Secretory Compartments

#### 2.1.1. α-Granules

α-Granules are the predominant storage organelles in platelets, housing a varied array of proteins that play roles in coagulation, inflammation, angiogenesis, and tissue remodeling. Principal components comprise platelet factor 4 (PF4/CXCL4), P-selectin, vascular endothelial growth factor (VEGF), platelet-derived growth factor, fibrinogen, and amyloid precursor protein (APP). Following platelet activation, α-granule release swiftly modifies the local microenvironment by facilitating leukocyte recruitment, endothelial activation, and extracellular matrix interactions [43,44]. Several components of α-granules are directly pertinent to neurodegenerative disease [45]. PF4 and P-selectin facilitate the formation of platelet-leukocyte aggregates and the activation of microglia, thus exacerbating neuroinflammatory cascades [46]. VEGF, traditionally recognized for its angiogenic properties, can enhance blood–brain barrier (BBB) permeability in pathological states, potentially allowing peripheral inflammatory mediators to infiltrate the central nervous system (CNS) [47]. The presence of APP in α-granules indicates that platelets may directly engage in amyloid-related processes, especially in AD, where aberrant APP processing and Aβ accumulation are key pathological characteristics [45,48].

#### 2.1.2. Dense Granules

Dense granules contain small-molecule mediators, including serotonin, ADP/ATP, calcium ions, and polyphosphates, allowing platelets to swiftly modulate vascular tone, hemostasis, and cellular signaling [49]. Serotonin released from dense granules functions as a powerful vasoactive and neuromodulatory agent, affecting vasoconstriction, synaptic transmission, and neurovascular coupling [50,51,52]. ADP and ATP function as pivotal purinergic signaling molecules, enhancing platelet activation and simultaneously influencing neurons, astrocytes, and microglia via P2 receptor interaction [53,54]. In the realm of neurodegeneration, excessive or dysregulated release of dense granules may lead to neuronal excitotoxicity and persistent neuroinflammation [55,56]. Increased extracellular ATP can activate microglial inflammasome pathways, while disrupted serotonin signaling has been associated with synaptic dysfunction and mood disorders commonly seen in neurodegenerative diseases [57,58].

#### 2.1.3. Lysosomes

Platelet lysosomes house proteases, glycosidases, and various degradative enzymes that facilitate extracellular matrix remodeling and maintain barrier integrity [59,60]. Despite being less thoroughly characterized than α- or dense granules, platelet lysosomal secretion may affect BBB integrity during inflammatory or thrombotic conditions. Activated platelets release lysosomal enzymes that may aid in the remodeling of the extracellular matrix and the degradation of tight junction components, potentially worsening neurovascular dysfunction and promoting leukocyte infiltration into the CNS [61,62,63]. Nonetheless, direct mechanistic evidence connecting platelet lysosomal secretion to BBB disruption in AD is currently insufficient and necessitates additional research.

### 2.2. Extracellular Vesicles as Long-Range Platelet Messengers

In addition to granule-mediated secretion, platelets release EVs that function as mobile carriers of molecular information. pEVs encompass microparticles, also known as microvesicles and exosomes, which vary in size, biogenesis, and cargo composition [64,65]. Microparticles are produced by outward budding of the plasma membrane during platelet activation, while exosomes derive from intraluminal vesicles within multivesicular bodies. pEVs encompass a multifaceted array of proteins, lipids, and nucleic acids, including integrins (e.g., α_IIb_β_3_), adhesion molecules, SNARE proteins, phosphatidylserine, and regulatory microRNAs (miRNAs). MiRNAs, particularly miR-223 and miR-126, are significantly abundant in pEVs and are associated with the regulation of endothelial integrity, inflammatory signaling, and neuronal survival [66,67,68,69]. pEVs functionally amplify the effects of platelet activation beyond the immediate vascular environment. Through their interaction with endothelial cells, leukocytes, and neural cells, EVs facilitate platelets’ involvement in long-distance communication and systemic adaptation [70,71]. Unlike granule release, which facilitates swift and localized effects, EVs offer a means for prolonged modulation of remote tissues, including the neurovascular unit [31,72].

**Table 1:** Platelet Secretory Compartments and Biological Functions in Neurodegenerative Diseases.

Secretory System	Subtype	Component	Function (Neurodegenerative Disease)	Ref.
Granules	α-granule	PDGF, fibrinogen, APP	Vascular inflammation, BBB integrity, Aβ handling, amyloid processing	[73,74]
		VEGF	BBB permeability, endothelial destabilization	[75,76]
		PF4(CXCL4), P-selectin	Microglia activation, Tau/Aβ pathology enhancement	[77,78]
	dense-granule	ATP/ADP, Ca^2+^	Neurotransmission, vasoconstriction, neuroinflammation, excitotoxicity	[79,80]
		serotonin	Synaptic signaling imbalance, neurovascular coupling	[81,82]
	lysosome	proteases, glycosidases	Extracellular matrix remodeling, BBB disruption	[83]
Extracellular vesicles	PMVs/MPs	miRNAs (miR-22, miR-126)	Regulation of inflammation endothelial integrity	[84,85]
		Proteins (SNAREs, integrins, adhesion molecules)	EV uptake, vascular inflammation, immune activation	[86]
		Lipids (phosphatidylserine)	Procoagulant signaling, neurovascular dysfunction	[87]
	exosome	miRNAs, signaling proteins	Long-range neurovascular communication	[88,89]

## 3. Integrated Roles of Platelet-Derived Factors in Alzheimer’s Disease

Increasing evidence indicates that platelet hyperactivation plays a pivotal role in influencing the neurovascular and inflammatory environment of AD [90]. Platelets from AD patients exhibit increased sensitivity to amyloid-β (Aβ), oxidative stress, and thrombin, leading to pronounced dysregulation and EV release [91,92,93]. These secretory responses establish platelets as primary enhancers of BBB, microglial activation, and pathological protein aggregation [94,95,96]. Instead of merely functioning as passive participants, platelets actively restructure the neurovascular unit, fostering a conducive environment for chronic neurodegeneration [97].

### 3.1. α-Granule-Derived Mediators

α-Granule-derived mediators establish a direct mechanistic connection between vascular dysfunction and neuronal pathology in AD [98]. PF4 and P-selectin play central roles in initiating platelet-leukocyte aggregation and promoting adhesion to cerebral endothelial cells through receptor-mediated interactions, including binding of P-selectin to P-selectin glycoprotein ligand-1 (PSGL-1) on leukocytes [46,99,100]. These interactions enhance leukocyte recruitment and sustain microglial activation within the neurovascular unit. Activated microglia subsequently release pro-inflammatory cytokines, ROS, and complement components that directly damage neurons and disrupt synaptic networks [101,102].

Platelet-derived PF4 further modulates microglial phenotype towards a pro-inflammatory state through activation of intracellular signaling pathways associated with tau kinase regulation, including glycogen synthase kinase-3β (GSK-3β) and cyclin-dependent kinase 5 (CDK5), thereby promoting tau hyperphosphorylation and cytoskeletal instability [103,104]. In parallel, inflammatory mediators derived from platelet activation enhance amyloidogenic processing of APP, contributing to increased Aβ accumulation and plaque formation [105].

VEGF, released from activated platelets, exerts potent effects on endothelial barrier function through activation of VEGFR2-dependent signaling pathways [106,107,108]. Sustained VEGF signaling induces cytoskeletal rearrangement and disruption of tight junction proteins, including claudin-5 and occludin, thereby increasing paracellular permeability of the BBB. This vascular permeability facilitates the infiltration of peripheral immune mediators, deposition of fibrinogen, and extravasation of plasma protein, which further activate microglia and astrocytes [109,110]. Fibrinogen accumulation within the brain parenchyma interacts with Aβ aggregates, amplifying inflammatory signaling and neuronal toxicity [111]. Collectively, these interconnected receptor-mediated and intracellular signaling events establish a feed-forward cycle of vascular impairment, neuroinflammation, and protein aggregation that accelerates neurodegenerative progression in AD.

### 3.2. Dense Granule-Derived Mediators

Neurotransmitters and nucleotides derived from platelet dense granules contribute to neuronal stress and glial activation through coordinated purinergic and serotonergic signaling pathways in AD [97]. Dysregulated serotonin release alters cerebral blood flow regulation and impairs synaptic plasticity through serotonergic receptor-mediated signaling, leading to early cognitive dysfunction [112,113]. Serotonergic imbalance additionally influences microglial reactivity, thereby amplifying inflammatory tone within the neurovascular unit [114]. Extracellular ATP and ADP released from activated platelets function as potent danger-associated molecular signals that activate purinergic receptors, including P2X7 and P2Y receptors, on microglia, astrocytes, and neurons [55,115,116]. At the molecular level, activation of P2XY receptors promotes assembly of the NOD-like receptor pyrin domain-containing 3 (NLRP3) inflammasome complex, leading to caspase-1 activation and subsequent secretion of interleukin-1β (IL-1β), thereby reinforcing neuroinflammatory signaling pathways. Sustained purinergic receptor activation further enhances intracellular calcium influx, triggering mitochondrial dysfunction and excitotoxic neuronal apoptosis [117,118]. The calcium-dependent processes intersect with oxidative stress signaling pathways, including activation of NADPH oxidase, resulting in excessive ROS generation and redox imbalance in AD brains [119]. Elevated ROS levels destabilize synaptic proteins and impair neurotransmission, thereby intensifying synaptic loss and neuronal degeneration, particularly in conjunction with Aβ-mediated toxicity [120,121].

Beyond inflammasome-driven responses, purinergic signaling also induces transcriptional and metabolic alterations that sustain neuronal vulnerability. Activation of purinergic receptors has been associated with downstream regulation of transcription factors such as activator protein-1 (AP-1) and nuclear factor of activated T cells (NFAT), which promotes sustained inflammatory gene expression in microglia and astrocytes [122,123]. These transcriptional responses enhance inducible nitric oxide synthase (iNOS) activity and nitric oxide production, further exacerbating oxidative and nitrosative stress within the neuronal microenvironment [124,125].

In parallel, prolonged nucleotide signaling disrupts neuronal metabolic homeostasis by impairing mitochondrial respiration and reducing ATP production efficiency [126,127]. Energy deficits resulting from mitochondrial dysfunction compromise synaptic vesicle recycling and neurotransmitter release, ultimately weakening synaptic maintenance mechanisms and accelerating dendritic spine loss [128,129]. The metabolic instability enhances neuronal susceptibility to excitotoxic injury and contributes to progressive synaptic remodeling observed during early neurodegenerative stages. Collectively, these interconnected receptor-mediated, transcriptional, and metabolic pathways position dense granule secretion as a critical driver of synaptic dysfunction, neuroinflammatory amplification, and neuronal degeneration in AD.

### 3.3. Platelet-Derived Extracellular Vesicle Cargo

pEVs have emerged as pivotal mediators of intercellular signaling in AD, amplifying the effects of platelet activation beyond localized vascular environments [93,130]. Circulating pEV levels are markedly increased in patients with AD and correlate with vascular dysfunction, cognitive decline, and inflammatory biomarkers, indicating their active contribution to disease progression rather than representing passive by-products of platelet activation [131,132]. EV uptake by recipient cells is mediated through receptor-dependent endocytosis pathways involving integrins, phosphatidylserine-binding receptors, and scavenger receptors, enabling selective delivery of vesicular cargo into endothelial, immune, and neural cells [133,134,135]. These receptor-mediated interactions trigger intracellular signaling cascades that regulate endothelial permeability, cytoskeletal remodeling, and inflammatory gene expression, thereby establishing pEVs as dynamic regulators of neurovascular integrity (Table 2).

#### 3.3.1. EV Biogenesis and Selective Cargo Loading in AD

In the AD milieu, platelet hyperreactivity induced by Aβ, oxidative stress, and thrombin signaling enhances membrane budding and multivesicular body formation, facilitating increased EV release [64,130]. Importantly, EV cargo loading is not random but is regulated by stress-responsive pathways that selectively incorporate miRNAs, inflammatory proteins, and procoagulant lipids into vesicles [28,136]. Redox-sensitive signaling pathways and cytoskeletal remodeling influence EV composition, resulting in vesicles enriched with inflammatory mediators and endothelial-modulating miRNAs [137]. These selective loading processes generate vesicles capable of transmitting disease-specific molecular signals across the neurovascular unit.

#### 3.3.2. EV-Mediated Enhancement of Neuroinflammation

Upon internalization by microglia and astrocytes, pEV cargo alters inflammatory transcriptional programs that sustain neuroinflammatory signaling [138,139]. MiRNAs such as miR-223 and miR-155 enhance pro-inflammatory gene expression by suppressing negative regulators of cytokine signaling pathways, thereby reinforcing nuclear factor kappa B (NF-κB)-dependent inflammatory responses [140,141]. Concurrently, EV-associated proteins promote activation of NADPH oxidase, leading to increased ROS production and sustained inflammasome activation. These signaling events amplify cytokine release and maintain chronic neuroinflammatory cycles characteristic of AD pathology.

#### 3.3.3. pEVs and the Dynamics of Protein Aggregation

Beyond inflammatory signaling, pEVs actively contribute to the propagation of pathological protein aggregation [142]. The lipid bilayer of EV membranes provides specialized microdomains that facilitate nucleation and aggregation of Aβ oligomer [143]. In addition, oxidative stress induced by EV-associated signaling enhances activation of tau kinase, including GSK-3β, thereby promoting tau hyperphosphorylation and cytoskeletal destabilization. The capacity of pEVs to transport misfolded proteins or aggregation-promoting factors resembles prion-like propagation mechanisms that facilitate spatial spread of neuropathology within the brain [142,144].

#### 3.3.4. Systemic-Central Signaling Axis

A defining feature of pEV-mediated signaling is their ability to bridge peripheral and central pathological processes. Circulating pEVs function as systemic carriers that transmit inflammatory and procoagulant signals from peripheral vascular environments to the central nervous system [145,146]. These vesicles harbor disease-stage-specific molecular signatures that establish a bidirectional communication axis linking vascular dysfunction, immune activation, and neuronal degeneration. Through repeated cycles of EV release, uptake, and intracellular signaling, peripheral platelet activation can be translated into sustained neurovascular injury within the brain.

**Figure 2:**
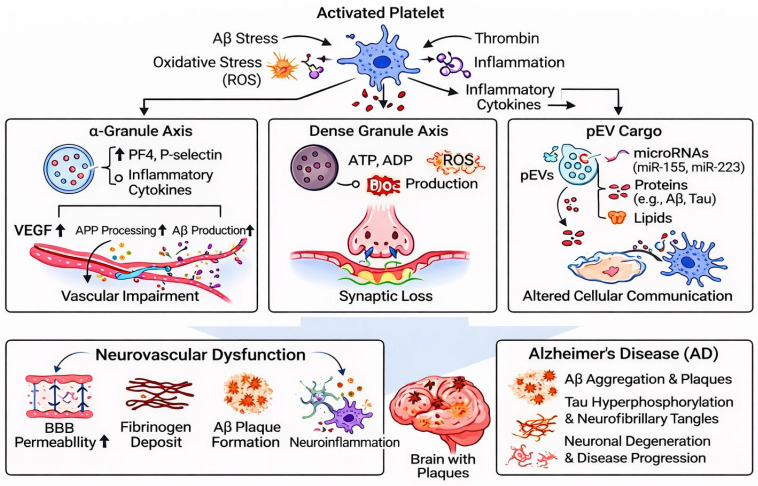
Mechanistic integration of platelet-derived mediators driving neurovascular dysfunction in AD.

Collectively, platelet-derived granule secretion and EV-mediated communication converge on a unified neurovascular axis characterized by BBB disruption, immune amplification, oxidative stress, and pathological protein accumulation. These coordinated mechanisms promote fibrinogen deposition, Aβ plaque formation, and sustained inflammatory signaling, ultimately driving progressive neurodegeneration in AD. These integrated signaling pathways are summarized schematically in Figure 2. Despite growing evidence supporting the role of platelet-derived EVs in neurodegenerative processes, several uncertainties remain. Many studies rely on cross-sectional human samples without longitudinal validation, limiting causal inference. Furthermore, variability in EV isolation methods introduces inconsistencies across datasets, complicating direct comparisons across studies [33]. Standardization of EV isolation protocols, quantitative normalization strategies, and validation in mechanistic animal models will be essential for translating EV-based findings into clinical actionable biomarkers and therapeutic targets.

**Table 2:** Platelet-derived factors in AD pathology.

EV Cargo	Representative Components	Primary Target Cells	Mechanistic Actions in AD	Pathological Consequences	Ref.
RNAs	miR-223	Microglia Endothelial cells	Regulates inflammatory gene expression, enhances ROS production	Chronic neuroinflammation, BBB dysfunction	[147]
	miR-126	Endothelial cells	Modulates vascular integrity signaling pathways	Increased BBB permeability	[85,148]
	miR-21	Microglia Astrocytes	Promotes pro-inflammatory activation	Sustained neuroinflammation	[149,150]
	miR-155	Immune cells	Amplifies inflammatory signaling	Accelerated neurodegeneration	[151,152]
Protein	Integrin α_IIb_β_3_	Endothelial cells Immune cells	Facilitates EV uptake and cell adhesion	Vascular inflammation	[153,154]
	P-selectin	Leukocytes	Enhances platelet-leukocyte interactions	Immune cell recruitment	[155,156]
	APP fragments	Neurons	Contributes to amyloidogenic processing	Aβ aggregation	[157,158]
	Pro-inflammatory cytokine proteins	Microglia	Activates inflammatory pathways	Neuronal injury	[159,160]
Lipid	Phosphatidylserine	Endothelial cells	Promotes procoagulant and inflammatory signaling	Neurovascular dysfunction	[161,162]
	Ceramides	Neurons, glia	Induces apoptosis and stress responses	Neuronal loss	[163,164]
	Oxidized phospholipids	Endothelium	Disrupts membrane integrity	BBB breakdown	[165,166]

## 4. Platelet-Derived Factors in Other Neurodegenerative Diseases

This review primarily focuses on AD, although platelet-derived mediators also play a role in the pathophysiology of other neurodegenerative diseases (Table 3).

### 4.1. Ischemic Stroke

Ischemic stroke is essentially a thromboinflammatory condition wherein platelet activation acts as both the initial catalyst for vascular occlusion and a significant enhancer of subsequent neurovascular damage [167,168]. Following cerebral vessel occlusion, platelets rapidly attach to exposed subendothelial matrix proteins, aggregate via integrin α_IIb_β_3_ interaction, and produce thrombin, resulting in clot formation and interruption of cerebral blood flow [169]. In addition to mechanical occlusion, activated platelets significantly influence the post-ischemic microenvironment by releasing granule-derived mediators and EVs that exacerbate inflammation, disrupt the BBB, and induce neuronal injury [170,171].

#### 4.1.1. Granule-Mediated Neurovascular Injury

The secretion of dense granules is pivotal in thrombotic propagation and excitotoxic injury subsequent to ischemia. ADP and ATP released from platelets enhance platelet recruitment via purinergic signaling while concurrently activating P2 receptors on neurons and glial cells [172]. Excess extracellular ATP activates microglial inflammasomes and induces calcium overload in neurons, worsening excitotoxic cell death [173]. Serotonin released from dense granules induces vasoconstriction of cerebral microvessels, thereby exacerbating perfusion deficits in peri-infarct regions and aggravating ischemic injury [174]. Platelet-derived α-granule mediators, including PF4, P-selectin, and VEGF, contribute to post-stroke inflammation and vascular permeability through mechanisms described in Section 3.1 [175,176]. In the ischemic microenvironment, these mediators amplify leukocyte recruitment and endothelial dysfunction, thereby reinforcing thromboinflammatory cascades and BBB disruption.

#### 4.1.2. pEVs as Mediators of Thromboinflammation

pEVs are significantly increased in the bloodstream after ischemic stroke and accumulate in cerebral microvasculature and infarcted tissue. These pEVs present phosphatidylserine and tissue factor, providing procoagulant surfaces that augment thrombin generation and fibrin deposition [177,178]. In addition to coagulation, pEVs convey inflammatory proteins, adhesion molecules, and regulatory miRNAs to endothelial and immune cells, exacerbating vascular dysfunction. The delivery of pro-inflammatory miRNAs and cytokine-associated cargo via pEVs facilitates endothelial activation, leukocyte adhesion, and oxidative stress [179]. Furthermore, pEVs engage directly with microglia and astrocytes, eliciting inflammatory gene expression and the formation of glial scar. Through these mechanisms, pEVs amplify the spatial and temporal effects of platelet activation beyond the initial thrombotic event, leading to secondary neurovascular damage and subsequent neuronal loss.

#### 4.1.3. Integrated Role in Ischemic Pathophysiology

Platelet granule secretion and EV release together initiate a thromboinflammatory cascade that connects vascular occlusion to advancing neurodegeneration. Although antiplatelet therapies significantly diminish the risk of recurrent stroke, their restricted effect on post-ischemic neuroinflammation suggests that researchers need to devise strategies aimed at platelet-derived inflammatory and EV-mediated signaling pathways [180].

### 4.2. Parkinson’s Disease

Parkinson’s disease (PD) is defined by the progressive degeneration of dopaminergic neurons in the substantia nigra, along with persistent neuroinflammation and vascular changes [181,182]. While traditionally regarded as a neurological disorder, mounting evidence suggests that peripheral immune and vascular elements, including platelets, play a role in disease progression.

#### 4.2.1. Granule-Derived Mediators and Neuroinflammation

Activated platelets in PD patients release α-granule-derived inflammatory mediators that enhance leukocyte recruitment and microglial activation through mechanisms similar to those described in AD (Section 3.1) [97]. Increased serotonin levels affect dopaminergic signaling and vascular tone, possibly leading to the dysregulated cerebral perfusion seen in PD [183,184]. VEGF released from α-granules may initially facilitate vascular repair; however, under chronic inflammatory conditions, it contributes to BBB permeability and leukocyte infiltration [47,182]. The compromised integrity of the barrier permits peripheral immune mediators to infiltrate neural tissue, thereby exacerbating neuroinflammatory cascades.

#### 4.2.2. pEVs and α-Synuclein Propagation

pEVs have emerged as potential carriers of α-synuclein, the pathogenic protein implicated in PD. α-Synuclein associated with pEVs can be transmitted between cells, facilitating misfolding and aggregation in recipient neurons and glial cells. The dissemination mediated by pEV resembles the prion-like propagation mechanisms suggested for the progression of PD [185]. Furthermore, pEV cargo comprises inflammatory proteins and regulatory miRNAs that influence microglial activation and oxidative stress responses. By selective absorption by neural cells, pEVs may exacerbate mitochondrial dysfunction and increase neuronal susceptibility.

#### 4.2.3. Crosstalk Between Vascular and Immune Systems

Platelet-mediated vascular inflammation in PD contributes to microvascular dysfunction and diminished cerebral perfusion, potentially intensifying neuronal stress. The synergistic effects of granule-derived mediators and EV signaling establish platelets as systemic amplifiers of neurodegenerative processes rather than mere passive participants.

### 4.3. Multiple Sclerosis

Multiple sclerosis is an autoimmune demyelinating disorder marked by recurrent breakdown of the BBB, leukocyte infiltration, and persistent neuroinflammation. Platelets actively participate in the initiation and maintenance of these pathological processes [186,187].

#### 4.3.1. Granule Secretion in the Recruitment of Immune Cells

Upon activation, platelets release α-granule-derived chemokines such as PF4 and RANTES, which attract T cells and monocytes to inflamed cerebral vessels [188,189]. P-selectin expression enhances platelet–leukocyte adhesion, thereby facilitating transendothelial migration into CNS tissue. These interactions are essential for the development of inflammatory lesions typical of multiple sclerosis [190]. ATP derived from dense granules further activates immune cells through purinergic receptors, augmenting cytokine production and inflammatory signaling within the neurovascular compartment [191].

#### 4.3.2. EV-Mediated Immune Modulation

pEVs are abundant in pro-inflammatory proteins and miRNAs that modulate T cell activation and endothelial permeability. These EVs augment the expression of adhesion molecules on endothelial cells, thereby facilitating leukocyte migration across the BBB [192,193]. Platelet activation occurs prior to the onset of clinical disease in experimental autoimmune encephalomyelitis, highlighting a pathogenic role for platelets in neuroinflammation. While direct evidence for pEV involvement is limited, EV-mediated mechanisms may facilitate immune activation and neurovascular dysfunction, ultimately leading to demyelination [187].

#### 4.3.3. Contribution to Chronic Neurovascular Dysfunction

By continuous granule secretion and EV release, platelets maintain a pro-inflammatory vascular milieu that facilitates persistent immune infiltration and neural injury. Targeting platelet-derived pathways may thus serve as a complementary therapeutic strategy in conjunction with immunomodulatory treatments.

**Table 3:** Comparative mechanisms of platelet-derived granules and EVs in neurological disorders.

Disease	Platelet Activation Trigger	Granule-Derived Mediators	EV Cargo	Neurovascular Effects	Pathological Outcome	Ref.
Alzheimer’s disease	Chronic inflammation Aβ exposure	PF4, P-selectin, VEGF, serotonin, ATP	MiR-223, 126, tau-related proteins	BBB disruption, microglia activation	Neuroinflammation, tau/Aβ pathology	[88,194]
Ischemic stroke	Shear stress, thrombin, hypoxia	ADP/ATP, serotonin, VEGF, PF4	Procoagulant EVs, inflammatory miRNAs	Thrombosis, BBB breakdown	Infarction, excitotoxic injury	[80,195]
Parkinson’s disease	Oxidative stress, inflammation	PF4, serotonin, VEGF	α-synuclein-containing EVs	Microvascular dysfunction	Protein aggregation, neuron loss	[196,197]
Multiple sclerosis	Immune activation	PF4, RANTES, ATP	Immune-modulatory EV miRNAs	Leukocyte BBB infiltration	Demyelination	[190]

## 5. Clinical and Translational Implications

Platelet-derived secretory products and EVs constitute a distinctive interface between vascular biology and neurodegenerative pathology, presenting innovative prospects for disease diagnosis, monitoring, and therapeutic intervention [42,198]. In contrast to CNS biomarkers that necessitate invasive cerebrospinal fluid extraction, platelet-derived mediators are prevalent in peripheral blood, facilitating minimally invasive evaluation of neurovascular dysfunction and inflammatory status [199,200]. Moreover, the mechanistic role of platelets in disease progression designates them as both indicators and regulators of neurodegenerative processes.

### 5.1. Platelet-Derived Factors as Biomarkers

The pursuit of dependable blood-based biomarkers for neurodegenerative diseases has intensified owing to the limitations of neuroimaging and cerebrospinal fluid analyses [199,201]. Platelets and their secretory products provide a biologically relevant and accessible biomarker source indicative of vascular inflammation, immune activation, and neurovascular unit integrity [55,99,198]. Soluble P-selectin and PF4 are well-established markers of platelet activation and are associated with systemic inflammatory burden [54,100]. Increased circulating levels of these mediators have been reported in patients with AD, ischemic stroke, and multiple sclerosis, associating platelet activation with disease severity and progression [202,203,204]. Serotonin, primarily contained in platelet dense granules, demonstrates modified plasma concentrations in neurodegenerative disorders and correlates with cognitive impairment and mood disturbances [58,194]. pEVs augment biomarker specificity through the encapsulation of disease-relevant cargo [40]. EV-associated miRNAs, including miR-223 and miR-126, modulate endothelial function, inflammatory signaling, and neuronal survival pathways, with their circulating levels exhibiting disease-specific alterations [205,206,207]. Proteomic profiling of pEVs has revealed inflammatory mediators, oxidative stress-associated proteins, and aggregation-prone peptides that reflect pathological processes in the CNS [179,208]. Significantly, EV cargo reflects dynamic disease states, enabling longitudinal monitoring of neurodegenerative progression and therapeutic efficacy [209,210,211]. This temporal sensitivity establishes platelet-derived biomarkers as potent instruments for early diagnosis and precision medicine approaches [198,212].

### 5.2. Therapeutics Targeting of Platelet-Derived Factors

Given their pivotal role in neurovascular dysfunction and inflammation, platelet secretory mechanisms constitute promising therapeutic targets [170,213]. Conventional antiplatelet treatment, such as aspirin and P_2_Y_12_ receptor inhibitors, diminishes thrombotic risk in ischemic stroke and exhibits limited protective effects against cognitive impairment [214,215]. Nonetheless, extensive inhibition of platelet aggregation carries bleeding risks and may inadequately mitigate platelet-mediated inflammatory signaling [216,217]. Focusing on granule secretion presents a more refined strategy [218]. Inhibitors of platelet degranulation pathways may attenuate the release of pro-inflammatory mediators while preserving essential hemostatic function [170]. Modulation of α-granule-derived VEGF signaling may stabilize BBB integrity, while regulating dense granule ATP/serotonin release could diminish excitotoxic and inflammatory cascades [219,220]. Oxidative stress pathways, particularly NADPH oxidase-dependent platelet activation, have been identified as upstream regulators of platelet secretion and EV release [141,221]. Pharmacological NADPH oxidase inhibition and antioxidant strategies may mitigate platelet-induced neurovascular damage by reducing ROS-mediated activation [222,223]. EV-targeted therapies represent an emerging frontier. Strategies include inhibiting EV biogenesis, blocking EV uptake by target cells, or engineering EV cargo to deliver neuroprotective molecules [224]. Modified pEVs may be utilized as natural nanocarriers for anti-inflammatory miRNAs or therapeutic proteins, capitalizing on their intrinsic biocompatibility and targeting ability [27,200]. Emerging therapeutic strategies targeting platelet-mediated inflammation are currently under investigation. In addition to conventional antiplatelet agents, novel pharmacological approaches targeting P2Y12 signaling, inflammasome activation, and oxidative stress pathways are being explored in preclinical and early clinical studies [225]. Furthermore, EV-based diagnostics are being actively evaluated as minimally invasive tools for disease monitoring and patient stratification. Several ongoing clinical studies are evaluating the impact of antiplatelet and anti-inflammatory interventions on cognitive decline and vascular dysfunction, highlighting the growing interest in platelet-targeted therapeutic strategies.

### 5.3. Liquid Biopsy Approaches Using Extracellular Vesicles

Liquid biopsy technologies utilizing circulating EVs are transforming disease diagnostics across oncology and cardiovascular medicine and are increasingly applied to neurodegenerative disorders [226,227]. pEVs are notably appealing because of their abundance, stability, and cargo profiles that respond to diseases [40,228]. Advanced isolation techniques combined with high-throughput omics platforms enable the comprehensive characterization of EV protein, lipid, and RNA content from small plasma volumes [229,230]. These approaches allow identification of disease-specific EV signatures indicative of neurovascular dysfunction, inflammatory activation, and protein aggregation processes [231,232]. Longitudinal liquid biopsy profiling of pEVs provides unprecedented opportunities to track disease progression, stratify patients, and monitor therapeutic efficacy [211,233]. Furthermore, the integration of EV biomarkers with clinical parameters and imaging data may facilitate early identification of neurodegenerative disease prior to overt neurological decline [234]. Challenges remain in standardizing EV isolation, normalization strategies, and cargo quantification [33,235]. Rapid technological advancements are accelerating the clinical translation of EV-based diagnostics, establishing pEVs as central components of future precision neurology frameworks [236].

### 5.4. Knowledge Gaps and Limitations in Platelet-Mediated Neurodegeneration Research

Despite growing recognition of platelet-derived mediators and EVs as contributors to neurodegenerative processes, several important knowledge gaps remain. Many clinical studies investigating platelet activation in neurodegenerative diseases rely on cross-sectional human cohorts, limiting the ability to establish causal relationships between platelet dysregulation and disease progression. Furthermore, variability in platelet isolation procedures and EV characterization methods introduces methodological inconsistencies across studies, complicating comparison or results. Another significant limitation arises from confounding factors commonly present in aging populations. Neurodegenerative disease cohorts frequently include individuals with cardiovascular disease, diabetes, chronic inflammation, and other systemic conditions known to independently alter platelet reactivity. In addition, widely prescribed medications such as antiplatelet agents, statins, and anti-inflammatory drugs can substantially influence platelet activation profiles and EV release patterns. These variables complicate interpretation of platelet-derived biomarkers and necessitate careful study design and stratification strategies.

At the mechanistic level, the precise molecular pathways linking platelet activation to neuronal dysfunction remain incompletely defined. While emerging evidence supports roles for purinergic signaling, oxidative stress pathways, and receptor-mediated EV uptake, many downstream signaling cascades have yet to be fully characterized in vivo. In particular, the extent to which platelet-derived EVs directly transport pathological cargo across the BBB remains an area of active investigation. Future research should prioritize standardized platelet phenotyping protocols, longitudinal cohort studies, and integration of multi-omics approaches, including proteomics, lipidomics, and single-cell transcriptomics. These efforts will be essential to clarify the causal role of platelet-derived factors in neurodegeneration and to support the development of clinically actionable biomarkers and therapeutic targets.

## 6. Conclusions

Platelets are increasingly recognized as multifunctional regulators that extend far beyond their classical hemostatic role. This review highlights how platelet-derived secretory systems—including α-granule release, dense granule secretion, and EV signaling—collectively function as integrated mediators of neurovascular dysfunction in neurodegenerative disease, particularly AD. Several key mechanistic insights emerge from this synthesis. First, α-granule-derived mediators promote endothelial activation, leukocyte recruitment, and BBB disruption, establishing a vascular environment that facilitates sustained neuroinflammation. Second, dense granule-derived nucleotides and neurotransmitters activate purinergic signaling pathways that amplify inflammasome activation, oxidative stress, and neuronal vulnerability. Third, pEVs extend these effects beyond local vascular sites by transporting bioactive cargo that modulates endothelial integrity, glial activation, and pathological protein aggregation. Together, these interconnected mechanisms support the concept that platelet secretory pathways function not as isolated processes but as coordinated amplifiers of neurovascular and neuroinflammatory signaling.

Importantly, this review proposes a unified neurovascular framework in which platelet granule secretion and EV-mediated communication converge to bridge peripheral vascular activation with central neurodegenerative progression. By integrating these traditionally separated mechanisms into a single conceptual axis, this framework provides a clearer mechanistic understanding of how systemic inflammatory and vascular signals propagate into neuronal injury and cognitive decline. Despite significant advances, several critical research priorities remain. Future studies should prioritize: (1) mechanistic validation using longitudinal animal models to establish causal relationships between platelet-derived mediators and neurodegenerative progression; (2) standardization of EV isolation, characterization, and quantification methods to improve reproducibility across studies; (3) integration of multi-omics approaches, including proteomics, transcriptomics, and single-cell analyses, to define disease-stage-specific platelet signatures; and (4) translational investigations assessing platelet-derived granules and EV cargo as predictive biomarkers and therapeutic targets in clinical settings.

Collectively, advancing mechanistic resolution and translational validation of platelet-derived signaling pathways will be essential for transforming platelet biology from a descriptive framework into a clinically actionable platform. These efforts may ultimately enable the development of targeted strategies that mitigate neurovascular dysfunction and slow the progression of neurodegenerative diseases.

## Data Availability

No new data were created or analyzed in this study.

## Abbreviations

The following abbreviations are used in this manuscript:

AD	Alzheimer’s disease
APP	Amyloid precursor protein
Aβ	Amyloid-β
ATP	Adenosine triphosphate
ADP	Adenosine diphosphate
BBB	Blood–brain barrier
CNS	Central nervous system
CDK5	Cyclin-dependent kinase 5
EV	Extracellular vesicle
GSK-3β	Glycogen synthase kinase-3 beta
IL-1β	Interleukin-1 beta
MiRNA	MicroRNA
NF-κB	Nuclear factor kappa B
NLRP3	NOD-like receptor pyrin domain-containing 3
PD	Parkinson’s disease
PF4	Platelet factor 4
pEV	Platelet-derived extracellular vesicle
P2X7	Purinergic receptor P2X7
P2Y12	Purinergic receptor P2Y12
RANTES	Regulated upon Activation, Normal T cell Expressed and Secreted
ROS	Reactive oxygen species
VEGF	Vascular endothelial growth factor
